# Feeding morphology and body size shape resource partitioning in an eared seal community

**DOI:** 10.1098/rsbl.2022.0534

**Published:** 2023-03-08

**Authors:** Ana M. Valenzuela-Toro, Rita Mehta, Nicholas D. Pyenson, Daniel P. Costa, Paul L. Koch

**Affiliations:** ^1^ Department of Ecology and Evolutionary Biology, University of California Santa Cruz, Santa Cruz, CA 95060, USA; ^2^ Department of Paleobiology, National Museum of Natural History, Smithsonian Institution, Washington, DC 20560, USA; ^3^ Department of Paleontology and Geology, Burke Museum of Natural History and Culture, Seattle, WA 98105, USA; ^4^ Institute of Marine Sciences, University of California Santa Cruz, Santa Cruz, CA 95064, USA; ^5^ Department of Earth and Planetary Sciences, University of California Santa Cruz, Santa Cruz, CA 95064, USA

**Keywords:** functional morphology, foraging ecology, marine mammals, community structure, niche partitioning, body size

## Abstract

Body size and feeding morphology influence how animals partition themselves within communities. We tested the relationships among sex, body size, skull morphology and foraging in sympatric otariids (eared seals) from the eastern North Pacific Ocean, the most diverse otariid community in the world. We recorded skull measurements and stable carbon (*δ*^13^C) and nitrogen (*δ*^15^N) isotope values (proxies for foraging) from museum specimens in four sympatric species: California sea lions (*Zalophus californianus*), Steller sea lions (*Eumetopias jubatus*), northern fur seals (*Callorhinus ursinus*) and Guadalupe fur seals (*Arctocephalus townsendi*). Species and sexes had statistical differences in size, skull morphology and foraging significantly affecting the *δ*^13^C values. Sea lions had higher *δ*^13^C values than fur seals, and males of all species had higher values than females. The *δ*^15^N values were correlated with species and feeding morphology; individuals with stronger bite forces had higher *δ*^15^N values. We also found a significant community-wide correlation between skull length (indicator of body length), and foraging, with larger individuals having nearshore habitat preferences, and consuming higher trophic level prey than smaller individuals. Still, there was no consistent association between these traits at the intraspecific level, indicating that other factors might account for foraging variability.

## 1. Introduction

Body size and other morphological differences play major roles in resource partitioning among sympatric species, influencing the structure of communities [1,2]. Among marine tetrapods, body size and feeding morphology affect foraging dynamics [3–5]. Larger taxa can dive deeper and longer, display lower relative metabolic rates than smaller taxa [6–10] and can exploit a vaster diversity of prey by reaching greater depths. Skull traits can limit prey size and processing efficiency [4,11–14], further influencing foraging dynamics [15–19]. Few studies have quantified the relationship between body size, feeding morphology and foraging ecology in co-occurring marine tetrapods (e.g. [20–23]). While these studies revealed associations between size, feeding morphology and trophic level, no consistent trends among species were uncovered presumably because of the lack of a comprehensive assessment of the communities examined. These studies included taxa (e.g. cetaceans, penguins and seals) with disparate body sizes and life histories, and it is possible that taxon-specific evolutionary trade-offs may be confounding these results. Therefore, additional studies testing ecomorphological relationships in closely related sympatric species can illuminate the factors influencing the structure of marine communities.

**Figure 1.**
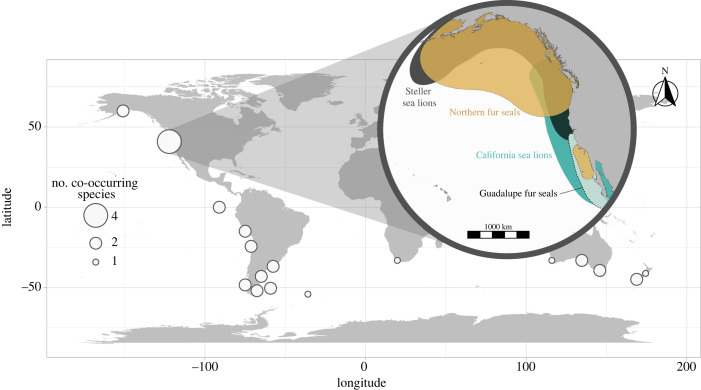
Distribution of otariids. Circles represent the location of major breeding colonies and their size, the number of coexisting species. Communities from sub-Antarctic islands are not depicted. Inset illustrates the distribution range of species inhabiting the eastern North Pacific Ocean. Modified from [24].

Pinnipeds (true seals, eared seals and walruses) are marine mammals that breed on land and forage in the water. Eared seals (otariids) are polygynous breeders that inhabit upwelling zones throughout the North Pacific and the Southern Hemisphere ([24]; figure 1). Otariids have been traditionally grouped into fur seals and sea lions based on morphological and foraging differences [25–30]. Sea lions have a larger body size, their insulation relies on a thick blubber layer and lactating females undertake short foraging trips. Fur seals, instead, are smaller in size, have a dense underfur coat that provides insulation, and females conduct long foraging trips. Nevertheless, fur seals and sea lions are not monophyletic (electronic supplementary material, figure S1), indicating repeated evolutionary convergence on these modes of life. They commonly co-occur throughout their range (figure 1), and variable levels of competition and resource partitioning have been described between them (e.g. [31–34]). Studies have shown that size and feeding functional morphology affect foraging performance in otariids (e.g. [11,27]), shaping these sympatric associations. However, the explicit association between size, feeding morphology and foraging in sympatric otariids, and their role in structuring their communities remain unknown.

We examined the association between body size, feeding morphology and bone collagen stable carbon (*δ*^13^C) and nitrogen (*δ*^15^N) isotopes, which are proxies of the foraging habitat and trophic level, respectively [35] in sympatric otariids from the eastern North Pacific Ocean. In this region, four species co-occur: California sea lions (*Zalophus californianus*), Steller sea lions (*Eumetopias jubatus*), northern fur seals (*Callorhinus ursinus*) and Guadalupe fur seals (*Arctocephalus townsendi*), constituting the most diverse otariid community in the world.

## 2. Methodology

### (a) Skull measurements and morphological indices

We measured 205 physically mature skulls (with fused bone sutures) collected from central and northern California between 1915 and 2015 from the sympatric Guadalupe fur seals (four females), northern fur seals (10 females, four males), California sea lions (53 females, 103 males) and Steller sea lions (22 females, nine males). We recorded five linear measurements of the skull using a digital caliper with an accuracy of 0.01 mm (electronic supplementary material, table S1). We also recorded standard body length (SL) of a subset of 141 specimens and used it to evaluate its relationship with skull length (condylobasal length; CBL). SL was recorded in the field by the original collectors and consisted of the straight-line distance from the snout to the tip of the tail. We calculated three morphological indices accounting for skull feeding mechanics. Mechanical advantage (MA) and skull shape index (SSI) served as proxies for the relative ability to generate bite force in the mandible and cranium, respectively, whereas the relative palatal length (RPL) indicated the relative size of the oral cavity.

### (b) Stable isotope analysis

We analysed the bulk *δ*^13^C and *δ*^15^N values of bone collagen of 205 specimens (representing a time-average from months to years). Samples consisted of approximately 20 mg of turbinate bone from the nasal cavity that were cleaned and demineralized [35]. Lipids were extracted by cycles of soaking and agitation in a petroleum ether solution followed by rinses with deionized water. Samples were freeze-dried and then weighed into tin capsules (Costech; 5 × 9 mm) for analysis. Isotope data are expressed in delta (*δ*) notation which for *δ*^13^C and *δ*^15^N (‰) = [(*R*_sample_/*R*_standard_) − 1] × 1000, where *R*_sample_ or *R*_standard_ are the ^13^C/^12^C and ^15^N/^14^N ratios in the sample or standard. Measurements were corrected to VPDB (Vienna PeeDee Belemnite) for *δ*^13^C and AIR for *δ*^15^N against an in-house gelatine standard reference material (PUGel) which is calibrated against international standard reference materials. Reports of instrument precision and reference materials are supplied in the electronic supplementary material. The atomic C : N ratio of samples ranged between 3.1 and 3.3, indicating well-preserved collagen [36].

### (c) Data analysis

We used R statistical software version 4.0.3 [37] for analyses. Normality and homoscedasticity of the data were inspected using diagnostic plots. The *δ*^13^C values were corrected by the Suess effect following [38] before analysis accounting for global decrease in the ^13^C concentration of atmospheric CO_2_ during the collection period. We further investigated the effect of temporal *δ*^13^C baseline changes by analysing specimens collected from 1990 onward in addition to the full dataset (electronic supplementary material). We conducted Spearman's correlation coefficient to test the relationship between CBL and SL and a two-way analysis of variance using species and sex as fixed variables and CBL and morphological indices as dependent variables to test differences between populations. We used generalized linear models (GLMs) to examine the drivers of the variability of the *δ*^13^C and *δ*^15^N values (response variables) using the function *glm*. Species, sex, CBL, MA, RPL and SSI were the explanatory variables. We verified the correlation between explanatory variables was less than 0.7 through the Pearson correlation coefficient using the packages *corrplot* [39] and *ggcorrplot* [40] (electronic supplementary material, figure S2). We ran models for *δ*^13^C and *δ*^15^N, employing a gamma distribution with an inverse link function and a Gaussian distribution for the absolute *δ*^13^C and *δ*^15^N values, respectively. We ranked models based on their Akaike's information criterion (AIC) using the package *AICcmodavg* [41]. The models with the lowest AIC values were considered to best fit [42]. Model validation was conducted by plotting residuals versus fitted values. We examined the community-wide relationship between the *δ*^13^C and *δ*^15^N values and the CBL using linear regression with sex as an explanatory variable, accounting for sexual dimorphism.

## 3. Results

CBL and SL were strongly correlated (*ρ* = 0.88, *p* < 0.001; electronic supplementary material, figure S3), indicating that skull length is a valid proxy of body size [43]. We found significant differences in the CBL between species (*F* = 804.90, *p* < 0.001) and sexes (*F* = 1691.38, *p* < 0.001), resulting in a size continuum from the smallest female northern fur seals (184.47 ± 10.00 mm) to the largest male Steller sea lions (371.44 ± 19.29 mm) (electronic supplementary material, table S2 and figure S4). Feeding morphology varied between species and sexes but no consistent differences were found (electronic supplementary material, figure S4 and table S2).

We used GLMs to examine the variability of the stable isotope composition. The selected GLM explained 50.1% of the *δ*^13^C variance (electronic supplementary material, table S3) and included the significant effect of species and sex. California (*t* = 3.43; *p* < 0.001) and Steller (*t* = 4.35; *p* < 0.001) sea lions had significantly higher *δ*^13^C values than coeval fur seals (electronic supplementary material, figure S3). Furthermore, males had significantly higher *δ*^13^C values than females (*t* = 2.25; *p* = 0.026). The additive effect of species, sex, CBL and morphological indices had a significant effect on the *δ*^15^N values with the model accounting for 34.0% of the variance (electronic supplementary material, table S3). Otariids overlapped in their *δ*^15^N values with only northern fur seals showing significantly lower *δ*^15^N values than sympatric species (*t* = −2.88; *p* = 0.0045). Still, we found that SSI had a significant effect on the *δ*^15^N values (*t* = 2.68, *p* = 0.0081) with individuals with stronger bite forces (as indicated by SSI) showing significantly higher values.

**Figure 2.**
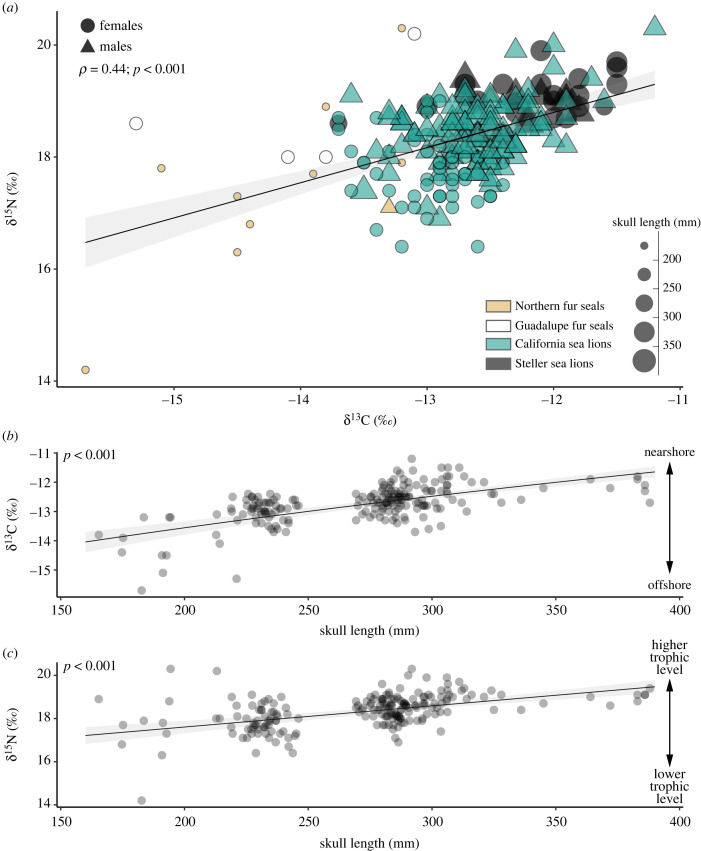
(*a*) Community-wide relationship between *δ*^13^C and *δ*^15^N values showing enrichment in larger skull sizes. Correlation between the skull length and *δ*^13^C (*b*) and *δ*^15^N values (*c*). Black lines represent the linear correlation model.

We obtained a significant community-wide correlation between the *δ*^13^C and *δ*^15^N values (*R*^2^ = 0.44, *p* < 0.001; figure 2*a*), with larger individuals generally occupying a higher position in the isotopic space. The GLMs showed a positive correlation between the CBL and the *δ*^13^C and *δ*^15^N values when accounting for sexual dimorphism (*δ*^13^C: *t* = 9.78, *p* < 0.001; *δ*^15^N: t = 0.68*, p* < 0.001; figure 2*b,c*). However, no significant relationship between the CBL and the *δ*^13^C and *δ*^15^N values was found at the intraspecific level. The only exceptions were female northern fur seals, with a positive relationship between *δ*^13^C and CBL, and male California sea lions, which had a positive relationship between CBL and both *δ*^13^C and *δ*^15^N values (electronic supplementary material, figure S5).

## 4. Discussion

Sympatric otariid species from the eastern North Pacific Ocean display significant differences in their size, feeding morphology and foraging ecology. California and Steller sea lions have larger body sizes than sympatric Galapagos and northern fur seals; however, no consistent differences in their feeding morphology were detected (electronic supplementary material, figure S4). Relative to fur seals, California and Steller sea lions were significantly ^13^C-enriched (1.2‰) with minimal differences in their *δ*^15^N values, hinting at foraging habitat differences (electronic supplementary material, figure S4). These results align with an offshore to nearshore ^13^C-enrichment gradient due to baseline differences linked with higher coastal primary productivity [44]. Sea lions might preferentially exploit nearshore habitats (with higher *δ*^13^C values) whereas fur seals would feed in offshore environments (with lower *δ*^13^C values), but on similar trophic level prey, resembling findings from animal-borne telemetry and dietary analyses (e.g. [45–50]). Still, significant latitudinal gradients on the isotope baselines exist along the California Current. Moreover, bone collagen turnover ranges from months to years, and its isotope composition reflects time-averaged ecological information. These factors and the different migratory patterns, complicate interpretations of *δ*^13^C and *δ*^15^N values variation among marine predators. Compound-specific stable isotopes on bone collagen and other tissues with shorter turnover rates will further clarify foraging differences among sympatric otariids.

The SSI, proxy for relative force production in the cranium, had a significant relationship with the *δ*^15^N values of sympatric otariids. Individuals with higher bite force capability tend to be more ^15^N-enriched than individuals with lower SSI, suggesting the preferential consumption of larger (and ^15^N-enriched) prey. Although pinnipeds do not masticate, consuming whole prey, otariids can chop large prey, breaking it into smaller pieces that are then consumed whole [51]. A stronger bite force would enable individuals to forage on larger prey [52]. The non-significant effect of the MA on the *δ*^15^N values suggests that bite force in sympatric otariids might have been achieved by alternative factors including shifts in muscle configuration.

Our study did not find a consistent association between body size and foraging ecology at the intraspecific level (electronic supplementary material, figure S5). While the low sample size can partially explain these results for some groups (e.g. Guadalupe fur seals), the lack of patterns in female and male California and Steller sea lions (with a larger sample size) suggests that, at the intraspecific level, morphological differences might be too small to drive differences in foraging. Although studies have found that body size influences foraging behaviour at the intraspecific level in some pinniped species (e.g. [10,53–55]), other studies have found no relationship (e.g. [56,57]). Recent research has emphasized how individual specialization emerging from physiological, behavioural and environmental trade-offs within populations can influence ecological dynamics, including small-scale resource competition [58–65]. Indeed, otariids display large individual behavioural variability independent from size or physical condition (e.g. [66–69]), suggesting that additional factors like ontogeny might account for foraging dynamics at the intraspecific level [70].

The community-wide association between body size and *δ*^13^C and *δ*^15^N values shows that larger individuals with stronger bite forces might forage closer to shore at an equivalent trophic level. This relationship can be explained by energetic trade-offs originating from benthic versus pelagic foraging (the predominant foraging strategies among otariids). Benthic diving entails longer durations and thus longer time spent at sea than pelagic foraging [71,72], making it more energetically costly [71,73]. Benthic and pelagic food webs are functionally and structurally different, influencing the energetic offset associated with their exploitation. Benthic food webs have higher species richness with a relatively homogeneous and predictable spatial distribution ([67] and references therein). Pelagic food webs have lower species diversity but more abundant and energy-dense in highly sporadic prey aggregations [69,74–76]. Larger individuals have a lower relative metabolic rate and cost of transport than smaller individuals (e.g. [10]), which combined with the consumption of a broader prey size range [77], likely enabled by their larger sizes and stronger bite capacities in coastal and benthic environments, might offset the higher absolute energetic costs of benthic diving. Smaller individuals have a smaller feeding apparatus, favouring the exploitation of schooling energy-rich but smaller pelagic fish [74–76].

Although phylogenetic relationships can constrain otariid ecology and morphology, shared ancestry is unlikely to explain our findings. Northern fur seals are the earliest diverging lineage of crown Otariidae [78]. By contrast, Guadalupe fur seals are nested within a southern clade, a derived group of fur seals, suggesting that the correspondence between the morphology and the isotope composition of these species emerged convergently. California and Steller sea lions together form a northern otariid clade [78] with larger body sizes and relatively higher *δ*^13^C and *δ*^15^N values than sympatric fur seals. Still, they display differences in feeding morphology and stable isotope composition, implying that factors distinct from phylogenetic relatedness might contribute to their foraging performance.

While we focused on otariids from the eastern North Pacific Ocean, the ecomorphological relationships found here may be prevalent in other geographical areas. Otariid communities throughout the Southern Hemisphere have lower taxonomic richness; however, comparable morphological and foraging disparities occur among sympatric species [24]. Likewise, the fossil record reveals that pinniped assemblages were diverse and had morphological differences analogous to modern communities [79,80]. These observations hint that variations in size and skull morphology among co-occurring pinnipeds have repeatedly evolved, contributing to resource segregation as in marine herbivore and terrestrial carnivore communities (e.g. [81–84]).

## Data Availability

All data and their description are available in the electronic supplementary material, files S2 and S3 [85].
